# Care‐full negotiation of hospital discharge and end‐of‐life care in an Indonesian palliative care unit

**DOI:** 10.1111/maq.70049

**Published:** 2025-12-17

**Authors:** Hanum Atikasari

**Affiliations:** ^1^ Institute of Cultural Anthropology and Development Sociology Leiden University Leiden, South Holland the Netherlands

**Keywords:** culturally sensitive communication, end‐of‐life care, hospital discharge, Indonesia, palliative care

## Abstract

How do palliative care professionals negotiate end‐of‐life care with family members when prognosis and dying are not openly discussed? Based on ethnographic fieldwork in an Indonesian palliative care unit, I argue that palliative care professionals employ implicit, ambiguous and culturally sensitive communication to carefully negotiate hospital discharge and discuss end‐of‐life care. I focus on listening to what is said and what remains unspoken in the embodied communicative practices about end‐of‐life care in family meetings to discuss hospital discharge. I show how palliative care professionals carefully navigate tensions between the hospital's need to discharge patients, family expectations of a cure, and the palliative care value of supporting patients and families. They do so by keeping the possibility of receiving treatment open while simultaneously using implicit language to suggest that end of life may be near.

## INTRODUCTION

“We are not talking about a cure [*sembuh*] anymore, but we are now focusing on getting healthy [*sehat*],” Dewi,^1^ the psychologist at the palliative care unit, told Yusuf, a former Islamic school teacher, and his two adult children during a family meeting. Yusuf's wife, Emi, had been diagnosed with stage IV breast cancer. She and her family had traveled from their hometown near Jakarta to this referral hospital located in the central part of Indonesia's capital, hoping she could be cured. Emi's medical treatment was covered by Indonesia's national health insurance. Since its introduction in 2014, advanced treatments have become increasingly available—though not always accessible—to previously uninsured and lower‐middle‐class patients, like Emi.

From the corner of the room, I saw Yusuf and his children looking puzzled. They were confused by the fact that Emi was being sent home as she had “not yet” (*belum*) received any treatment. Instead of talking to the oncologists about a treatment plan, Emi and her family had been referred to the palliative care unit to “prepare” her for hospital discharge.

Palliative care was a new specialty in the hospital and remains little known in Indonesia; Emi's family, like many others, was not familiar with it. Strikingly, before referring them to the unit, none of the treating specialists had informed Emi and her family of the illness's progression or her medical examination results, let alone her prognosis. The referral to palliative care meant that the palliative care team now had the mandate to support and care for Emi, whose condition was declining and who was likely no longer receiving curative treatment. The palliative care team needed to prepare Emi and her family for going home. However, they first had to explain to the family that Emi might not receive curative treatment at all.

In this tertiary public hospital, palliative care professionals often have to break bad news to patients and their families because the treating physicians have not done so. They may be reluctant to discuss prognosis and end‐of‐life issues,^2^ as they fear such conversations can be distressing for patients and their families (see also Martina et al., 2022). At the same time, palliative care professionals need to manage families’ and patients’ emotions and reactions to their referral to palliative care and their unexpected hospital discharge. These professionals find themselves in a rather tense situation because the patients and their family members are still hoping for treatment leading to a cure. In such cases, palliative care professionals strike a balance between sensitivity and communicating technical and medical knowledge. How, then, do palliative care professionals negotiate hospital discharge and end‐of‐life care with family members when prognosis and dying are not openly discussed?

Anthropologists have highlighted that patients and families may perceive direct communication about dying, or mentioning the patient's terminal diagnosis and prognosis, to have potentially harmful consequences (Arkin, 2020; Banerjee, 2020; Stonington, 2020; Samuels, 2023). For example, in his work on cancer care in Delhi, Dwaipayan Banerjee (2020) shows how revealing a cancer diagnosis can reshape social relationships in unexpected and disruptive ways. Religious beliefs may form another reason for avoiding discussions about prognosis, for example, when patients and relatives believe that death is determined by God and thus cannot be predicted by physicians (see e.g., Samuels, 2023).

Here, I argue that in a context where direct discussion of end of life is considered harmful or is culturally and religiously proscribed, implicit communication strategies may enable sensitive and carefully attuned engagement with end‐of‐life care. By implicit communication, I refer to conveying meaning indirectly through culturally shared cues, contextual framing, and linguistic subtleties, such as the use of euphemism. To support this argument, I build on existing anthropological literature on silence and moral and ethical affordance of language in the embodied constitution of care (Arkin, 2020; Black, 2015, 2018; Lambek, 2010; Samuels, 2023). As Steven Black has suggested, the embodied performance of linguistic practice “allows for indirection and engagement with multiple, sometimes conflicting, public discourse about morality” (2015, 248). While Black demonstrates how such performance helps HIV‐positive individuals in South Africa navigate dilemmas of disclosure and secrecy, in this paper, I explore how implicit language functions as a culturally situated communicative strategy in end‐of‐life care. I suggest that implicit language not only facilitates the negotiation of prognostic disclosure and sensitivity towards patients and their families to not openly discuss death and dying, but also plays a crucial role in dealing with the uncertainty that characterizes the dying process.

Below, I will show how palliative care professionals in Jakarta maintain a balance between medical knowledge, patient and family hope, and the institutional positioning of the palliative care unit within a health care system marked by expanding universal health coverage. They do this by keeping the possibility of receiving treatment open while concurrently implicitly talking end‐of‐life care through terms like *belum* (not yet) and shifting the narrative from *sembuh* (cure) to *sehat* (healthy), as psychologist Dewi did in the opening vignette, and as I will further discuss below.

This paper is based on ethnographic data from family meetings at the palliative care unit of the hospital where Emi was treated. These data were gathered as part of a larger ethnographic study on palliative care provision in Jakarta, conducted between 2022 and 2024, details of which will be discussed below. By exploring the palliative care professionals’ practices of implicit communication, drawing specifically on the example of communication with Emi's family, this paper also contributes to the anthropological literature on end‐of‐life care and palliative care. Despite the global expansion of palliative care and the growing importance of end‐of‐life care for individuals facing death around the world, these topics have received limited scholarly attention within medical anthropology (but see Zaman et al., 2017; Stonington, 2020; Arkin, 2020; Surawy‐Stepney, 2024; Cohn et al., 2024). Crucially, they have received even less attention in the Global South and particularly in Islamic majority countries. This includes Indonesia, the world's largest Muslim population, where over 87% of the citizens identify as Muslim and health care professionals often share a religious understanding with their patients.^3^ My analysis below suggests that in the context of the implementation of palliative care in a place where people were previously unfamiliar with the practice, and where prognosis is usually not openly discussed, palliative care workers navigate the tensions of communication in the end‐of‐life care discharge process in what can be called a ‘care‐full’ way, both careful and full of care (see also Goodwin, 2015).

Before discussing hospital discharge and palliative care in Indonesia and showing how palliative care professionals use implicit communication, I first outline how studies on embodied communication and care can inform an analysis of the care‐full negotiation of end‐of‐life care.

## EMBODIED COMMUNICATION AND CARE

Embodied communication, including through language, is an integral part of care. In recent years, embodied communicative acts, and particularly implicit communication and silences, have received growing attention from medical anthropologists (see Arkin, 2020; Black, 2015, 2018; Samuels, 2021; Shohet, 2021; Throop, 2010). This includes communication around cancer diagnosis (Banerjee, 2020; Livingston, 2012), HIV diagnosis (Black, 2015; Samuels, 2021), anorexia nervosa (Shohet, 2017), and end‐of‐life care (Arkin, 2020; Samuels, 2023; Stonington, 2020). Drawing on ethnographic research with a Zulu HIV support‐group choir in South Africa, Black (2015) shows how, through singing and performing in a choir, HIV‐positive individuals engage in communicating their HIV status, while also embodying a public discourse of morality by not disclosing anything about their condition directly. Black thereby highlights that disclosure is more than a discrete event. Rather, he posits, it should be understood as an ongoing process, and it is sometimes characterized by ambiguity—when and how to disclose it is based on the orientation of social duties. Disclosure is a communicative act, even if that act is implicit or implied (Black, 2015).

In the increasingly medicalized context of end‐of‐life care, death, and dying, open conversations about diagnosis and prognosis are often encouraged (see Kaufman, 2005). However, as social scientists have pointed out, physicians, including palliative care specialists, often face challenges when trying to communicate directly and openly about dying (Arkin, 2020; Olson et al., 2020; Stonington, 2020). Their studies show how health workers rely on emotional and cultural cues to tailor their communications to patients and their families. Olson et al. (2020), for example, highlight that end‐of‐life care communication involves reflexive emotional labor. Australian clinicians in their study navigated between professional protocols and individual family dynamics by letting patients and families lead the conversation. Rather than following scripted guidelines, they listened to emotional and linguistic cues and responded accordingly.

In addition, anthropologists have pointed out the importance of word choice in the context of end‐of‐life care, such as when negotiating the patient's options (Kaufman, 2005), preserving hope against all odds (Good et al., 1990), or “orchestrating” a good death in palliative care (Arkin, 2020). Patients and families may perceive particular words to produce a potentially harmful effect (Arkin, 2020; Banerjee, 2020; Stonington, 2020). In an ethnographic study conducted in a southern French hospital, where overwhelmingly non‐Muslim healthcare professionals were treating Muslim patients, Kimberly Arkin shows that the discursive content conveyed by palliative care professionals communicates that further treatment for the patient will not be effective, but the performance through which they do so is much more ambiguous. While some healthcare workers in her study criticized such lack of transparent communication, they also acknowledged that words were not—and might never be—clear or straightforward ways to communicate. They realized that words such as “diagnosis” and “prognosis” might cause distress for patients and families and could therefore become a potentially harmful performative act. Arkin concludes that by avoiding such words, healthcare professionals worked to create an “ethically acceptable death” (Arkin, 2020, 181).

In a rather different setting, Scott Stonington (2020) shows how interlocutors in Northern Thailand worry that mentioning patients’ terminal prognosis could make them lose their *kamlang chai* (heart–mind energy) and, therefore, actually speed up the dying process. Strikingly, the term *kamlang chai* is similar to what some of the healthcare workers and patients in Indonesia describe as keeping the life spirit (*semangat*). Concealing the prognosis or avoiding discussion of death and dying may thus, both in Thailand and Indonesia, be a way of “protecting” patients from losing this life spirit, and are subject to careful communication strategies of healthcare workers in a variety of settings.

## HOSPITAL DISCHARGE AND PALLIATIVE CARE IN INDONESIA

In many countries, including Indonesia, improvements in the accessibility and availability of medical care and technology have resulted in many more people accessing healthcare and hospitalization, including those with advanced cancer. Like in high‐income countries in the Global North, this has not only offered patients and their families hope for a cure but has also resulted in demands for “heroic intervention” to sustain life, particularly from patients’ relatives, who may be unable to let go of their loved ones (Kaufman, 2005, 97–102). As Sharon Kaufman has shown, this demand for intervention can continue until the family is finally “exhausted” and calls it quits. However, when curative treatment is exhausted, and there is a limit to insurance coverage for long‐term hospital care, patients have to be discharged from the hospital.^4^ This negotiation of hospital discharge can be challenging for patients and family caregivers, as well as healthcare workers, because of the perceived inappropriateness of openly discussing the end of life, and post‐discharge care is often uncertain. The organizational structure of the healthcare system also plays a significant role in the experiences of hospital discharge (see also Mattingly, 2010, 2011; Poulin et al., 2023; Skovgaard et al., 2022).

Increasingly, it is the discipline of palliative care that is mobilized to assist with the discharge process and discussions around end‐of‐life care (Broom et al., 2013; Cohn et al., 2024; Kirby et al., 2014). This is also the case in many countries in the Global South, where palliative care as part of global health interventions is expanding (Knaul et al., 2018; Zaman et al., 2017). In Indonesia, palliative care was first introduced in 1992, and subsequently the Ministry of Health issued a decree on palliative care services for patients with chronic illness in 2007, which was amended in December 2023 (Ministry of Health, 2007, 2023; Putranto et al., 2017). However, implementation has been slow and palliative care services are currently still mostly limited to tertiary hospitals in a few major cities, particularly on the island of Java.

The emerging palliative care services in Indonesia have also been influenced by the global palliative care movement, which is rooted in the modern hospice and palliative care movement that developed in the United Kingdom and the United States in the 1960s and 1970s. Most palliative care physicians in Indonesia receive training from countries where palliative care is a long‐developed specialty, such as the United Kingdom and Australia. In 2015, the Indonesian Cancer Foundation in Jakarta collaborated with the Singapore International Foundation to develop a 3‐year palliative care training program for physicians and allied health professionals from five national hospitals and seven district hospitals in Jakarta, including some of the palliative care workers I followed for this research. This initiative was one of the largest palliative care training programs in Indonesia. Participants learned about pain and symptom management, communication, psychosocial and spiritual support, and ethics (see also Eng et al., 2023).

In the tertiary hospital where I carried out my ethnographic research, specialists often refer patients with advanced serious illness, who will likely no longer receive curative treatment and need to be discharged from the hospital, to palliative care. In many cases, the ward nurse informs family members of the need to meet with the palliative care team before the patient is discharged from the hospital. Yet, as mentioned above, when they attend palliative care meetings, patients and family members have often not yet been informed of their illness's progression. Consequently, palliative care professionals have to carefully navigate discussions about end‐of‐life care, including being careful about what they do or do not say. The implicit disclosure that they practice aims to help patients’ families to manage their hope and expectations of (curative) hospital care.

## ISLAM, CULTURE, AND THE END OF LIFE IN INDONESIA

Religiosity and spirituality are central to everyday life in Indonesia, including in biomedical practices. Regardless of people's individual religious beliefs, spiritual and religious input are very common in daily communication. It is also common practice to use religious language when discussing palliative care (Rochmawati et al., 2018; Martina et al., 2022). For example, a physician mentioned in the study by Martina et al. (2022) used the term “*mudarat*,” which in an Islamic context means “harm” and is therefore prohibited, to help Muslim patients in Indonesia better understand the concept of futile treatment (as treatment that could “harm” the body) and to convince them that letting go of such futile treatment does not equal “giving up.”

Healthcare professionals in Indonesia often feel uncomfortable about discussing illness prognosis and dying (see also Martina et al., 2022; Samuels, 2023). In one of the family meetings I observed, Dr Ani, a palliative care specialist, refused to answer a question from a patient's family about the time the patient had left to live. She said (with her tone rising) that she did not like that sort of question. She noted that “while the patient's cancer presented as being in the late stage mentioned in many studies, which are predominantly conducted in Western countries, God's will might be different.” She emphasized it was “all in God's hands.” Elsewhere in Indonesia, health workers are also reluctant to discuss prognosis because they do not want to preempt what is determined by God; it would be (religiously) forbidden for physicians to predict the number of days, weeks, or years a patient has left to live (Martina et al., 2022). Moreover, many people believe it is prohibited, or in Javanese, *pamali*, to say out loud that someone is dying, as this could result in death occurring, and thus, many people avoid articulating the likelihood of death or putting it into words.

In Islamic everyday life, death is something that is often discussed, particularly when talking about the afterlife—as many Muslims believe that life is a stage of preparation for the hereafter (Al‐Shahri, 2016; Birchok, 2019; Othman et al., 2025). Yet, I have observed in Indonesia that many Muslim families tend to avoid discussions about the prospect of dying.^5^ The dying process is often discussed only in retrospect, after the person has passed away (see also Samuels, 2023; Seise, 2021). Family members rather emphasize the importance of maintaining faith and surrendering to God's will (*Ikhlas*) while continuing to seek curative treatment (see also Samuels, 2023; Henriks et al., 2012; Arkin, 2020).

Previous studies have similarly noted that many Muslims perceive illness as a divine “gift”—a test of faith through which the individual and their family are drawn closer to God, with the promise of reward in the afterlife for those who endure with patience (Al‐Shahri, 2016; Choong, 2015; Othman et al., 2025). While palliative care workers in Jakarta treat patients with diverse religions, Islamic concepts tend to generally shape the background of their practice. These usually resonate with adherents of other religions, reflecting the nation's longstanding belief in one God across diverse faiths. In the family meetings I attended, the spiritual worker Ahmed and sometimes the psychologist Dewi frequently invoked these Islamic concepts, weaving them into the conversation when negotiating hospital discharge.

In Indonesia, family plays an essential role in patient care, not only at the end‐of‐life stage but also throughout the care trajectory (for similar observations elsewhere in Southeast Asia, see Shohet, 2021; Stonington, 2020). In this context, family refers not only to the nuclear family or blood relations, but also to the extended family and those caring for the patient (Effendy et al., 2014). When someone is hospitalized, family members will visit and help patients continuously, often taking turns to stay with the patient in the hospital. In the absence of formal homecare options, strong family relationships, a pervasive sense of moral duty, and the reciprocal responsibility of care often ensure the availability of informal care after hospital discharge (Kristanti et al., 2019).

Similar to many countries in the Global South and many Muslim‐majority countries, family in Indonesia also plays a significant role in the medical decision‐making process and prognosis disclosure, and thus they are an important part of hospital discharge conversations (see also Martina et al., 2022; Effendy et al., 2014; Surawy‐Stepney, 2024; Ntizimira, 2023; Al‐Wamer & Downar, 2014). Family members often function as intermediaries in both collecting relevant information to facilitate patient care and discussing medical information with physicians, particularly in contexts where direct communication with the patient is mediated by kinship norms or cultural expectations (see e.g., Stonington, 2020; Surawy‐Stepney, 2024). In Indonesia, it is common for health workers to discuss the patients’ condition and (end‐of‐life) care with the family prior to talking to the patients themselves. Physicians often consult with family members to determine the most appropriate and careful way to disclose the progression of illness to the patient. This practice may differ from those commonly observed in Western medical settings, where direct communication regarding medical information and prognosis with the patient is typically emphasized.

## METHODS

This paper is based on a larger ethnographic research project on palliative care provision in Jakarta, Indonesia. As the capital of Indonesia, Jakarta, a densely populated urban environment with over 11 million residents, hosts a wide range of tertiary and specialized hospitals that attract patients from across the archipelago seeking advanced medical care. While these hospitals are located throughout the city, Jakarta's severe traffic congestion often makes accessing healthcare a significant challenge for those who, like Emi, live on the outskirts of the city.

In this study, data collection focused on the palliative care unit in a tertiary hospital and was conducted over 8 months in 2022.^6^ This public hospital is among the largest in Indonesia, offering advanced cancer treatment. It serves patients from a wide range of socioeconomic backgrounds, particularly following the introduction of the National Health Insurance (NHI) program. At the time of the fieldwork, over 90% of the patient care in this hospital was covered by NHI. This large, high‐rise referral hospital is equipped with advanced cancer treatment facilities. Palliative care services were introduced at the hospital in 1996. They remained under the medical rehabilitation department until approximately seven years prior to my arrival, when they were formally established as an independent unit. The unit takes a multidisciplinary approach, with a team that includes palliative care physicians, a neuro‐oncologist, an anesthesiologist, nurses, a psychologist, a social worker, and a spiritual worker.

During my fieldwork, I participated in the daily activities in the palliative care unit, interviewed palliative care professionals, and observed the interactions between palliative care professionals and family members in family meetings. I zoomed in on the role of palliative care professionals in the discharge of advanced‐stage cancer patients from the hospital to the home during family meetings. Such meetings are a space where palliative care professionals can share information on illness progression and prognosis and develop a care plan. Palliative care professionals invited me to attend these meetings, introducing me to the family members as a researcher. At this hospital, it is common practice for researchers, interns, and trainee physicians to observe and shadow healthcare activities. Following the introduction, I obtained verbal consent from the family members before proceeding.

To understand how end‐of‐life care was negotiated, I focused on the silences, (embodied) communicative acts, and language used in the meetings. In doing so, I used what Dragojlovic and Samuels (2023) have described as “attuned listening to silent reverberations,” an approach in which ethnographers reflect on their intersubjective engagement of being with and observing what resonates through and within silences. This resonance can potentially form another story or overlapping stories that may both align with and diverge from the established discourse, as I will show below. Paradoxically, it is through this close attention to narratives that we become attuned to what remains unspoken (Shohet, 2021). Attuned listening to silences is therefore a particularly useful method in understanding how end‐of‐life care is negotiated due to the sensitivity surrounding discussions of death, dying, and prognosis.

I documented my observations through brief fieldnotes taken during the meetings, followed by more detailed fieldnotes written afterward. Interviews were tape‐recorded and transcribed. All notes and quotations were originally written in Indonesian and subsequently translated into English. Using thematic analysis, I carefully examined the data from the 21 family meetings I attended and interviews with the palliative care professionals, reading through them multiple times with attention to themes, concepts and recurrent use of specific words to identify recurring patterns and themes.

Reflexivity was integrated throughout the research and analysis. As a native Indonesian speaker who was raised in Indonesia and lived in Indonesia for over 25 years, I am familiar with the implicit and nuanced use of language in everyday interactions, which informed my interpretation of the data. The analytical process was iterative, involving continuous reflection and refinement. I engaged in discussions with peers and presented preliminary findings in an end‐of‐life care workshop and a writing workshop to further develop and refine my thematic interpretations and to maintain analytical rigor by minimizing my implicit assumptions.

In the next section, I will show how palliative care professionals use silence and implicit communication to reduce the social tensions that arise from hospital discharge processes. I will particularly zoom in on the ways in which palliative care workers carefully disclose information about prognosis and end‐of‐life care to make the hospital discharge process more bearable. To do so, I will first return to the family meeting about Emi, the patient with stage‐IV breast cancer whom we met at the beginning of this article.

## CARE‐FULL NEGOTIATION OF END‐OF‐LIFE CARE AND HOSPITAL DISCHARGE

### Keeping the possibility of treatment open

In the meeting with Emi's family that I described at the beginning of this article, I joined Dewi, the psychologist at the palliative care unit, Dr Stefani (a palliative care physician), nurse Nina, spiritual worker Ahmed, and social worker Sari. Emi was not present, but her husband and two adult children were. At the beginning of the meeting, Dr Stefani asked Emi's family what they understood about Emi's condition and her illness. Yusuf, Emi's husband, explained Emi's care trajectory chronologically, from receiving the diagnosis to her current situation as an in‐patient at this tertiary hospital. His children sometimes added details to the story.

After having been at the hospital for almost a week, Emi had not received any treatment or examination results. Her family kept waiting and wondering when she would receive treatment. But instead of starting treatment, the oncologist had suddenly referred them to the palliative care unit to prepare Emi for hospital discharge. The ward nurse had informed Yusuf that his wife would be discharged from the hospital soon and that the family needed to have a meeting with the palliative care team before her discharge. Yusuf and his family did not understand the referral and wanted to know why Emi was being sent home while she was still ill and bedridden. As far as the family knew, Emi had had a tumor when she was admitted to the hospital, and her symptoms had only worsened, so they did not understand why she was now being discharged.

After listening to the story, Dr Stefani excused herself and went to the consultation room next door, taking her laptop. She had not expected that the family did not know anything yet about the severity of the patient's condition. A few minutes later, she returned and explained the CT scan and X‐ray results to the family. The family listened to her carefully and nodded occasionally while Dr Stefani pointed out the abnormalities in the scan results. For Yusuf and his children, it was the first time they had an opportunity to sit together and receive a long and clear explanation from a physician and healthcare team. The children also had the opportunity to ask the health professionals questions about their mother's condition. In the consultations they previously had, the family had limited time to interact with the specialists. Like many patients and family members whom I met during my fieldwork, especially those of lower socioeconomic status, Yusuf and his family said that they were hesitant to ask specialists questions. This hesitancy can result from medical hierarchy and social distance between the healthcare professionals, the patients, and their families, resulting in all the parties being very polite in order to maintain harmony and avoid conflict (see also Claramita & Susilo, 2024).

When Dr Stefani finished her explanation, Emi's son asked her, “What's next, doctor? When can my mother continue with the treatment?” Dr Stefani answered:

“Unfortunately, her condition is not very good. So, it is not yet possible (*belum bisa*) to continue with cancer treatment at this stage, as you can see from the scan. People like us, whose condition is ‘good,’ can end up in the emergency room after chemotherapy, let alone someone who is not in good condition. So, it is not that we do not want to give the patient treatment; it all depends on the patient's condition.”

The term *belum* (not yet) is widely used in the Indonesian language to soften the meaning of saying no. In this case, the term *belum* is an ambiguous language and saves the doctor from stating the bad news in a manner that is too direct. It is a way to maintain expectations and hope for the patients and their families. The term *belum* is also ambivalent because it implies that future treatment may still happen. By using *belum*, Dr Stefani implies that there might come a time when treatment will be possible. At the same time, though, she does not actually say that treatment will continue when the patient's condition is better. In some instances of discharge to palliative care, patients may indeed still receive treatment from their treating physician. Thus, for some, there is the possibility of readmission.

In a one‐on‐one interview I had with Dr Stefani, she mentioned that patients with advanced cancer treated at the hospital are “highly sensitive” due to the high level of stress they are experiencing as a result of their illness, which may have included undergoing treatment with many side effects. I often heard palliative care professionals who have the mandate to support such patients use euphemisms, such as *belum*, or phrases like *menunggu perkembangan* (“waiting for further developments”) and *masih dipertimbangkan* (“still under consideration”). This is a form of ambiguous language used to comfort patients and their family members and, particularly, to deal with the suddenness of the hospital discharge. It helped the family understand the seriousness of the illness and realize that even if there was a chance of further treatment, there was also a chance of no further treatment. In this way, health care workers carefully negotiate hospital discharge and end‐of‐life care by leaving open possibilities without being too definitive about the situation.

However, one of the palliative care staff with whom I spoke was critical of keeping the possibility of treatment open by saying *belum* (not yet). She suggested that the term *belum* may create the expectation and hope that treatment could be received post‐discharge, even though, in most cases, physicians know this is unlikely to happen. Patients will not return as their condition is declining. The term *belum* (not yet) may therefore contradict the discharge and the referral to palliative care. Nevertheless, most palliative care staff used this term, as they considered the potentially distressing effect of too direct information provisions more important. Below, I will explore a second example of how palliative care professionals use implicit language, namely through a narrative of shifting from focusing on being cured (*sembuh*) to being healthy (*sehat*).

### Not being cured but being healthy

As mentioned above, referral to the palliative care unit often creates confusion for families, due to their unfamiliarity with the specialty. In my observations, while some family members had searched for the term on the Internet prior to the family meeting with the palliative care unit and were terrified when they arrived because they were immediately confronted with the thought of death and dying, most families came with little prior knowledge. Therefore, palliative care professionals first needed to introduce their roles and the aim of palliative care. During the meeting with Yusuf and his two children, for example, Dr Stefani emphasized the role of palliative care, which focuses on comfort, quality of life, and symptom management.

“So, now, what is palliative care? Palliative care aims to ease suffering. We prioritize comfort and quality of life. We do not look at the disease but try to manage the symptoms. For instance, if the patient experiences pain or breathlessness, we will try to control those symptoms. So, we no longer use the term ‘cured’ (*sembuh*). We aim to make the patient feel comfortable.”

The psychologist, Dewi, who was sitting next to me during the meeting, added the words that are at the beginning of this paper:

“We are not talking about a cure (*sembuh*) anymore, but we are now focusing on her health and getting ‘healthy’ (*sehat*).”

There was a long pause before Dewi elaborated on what she meant by not being cured but “getting healthy.” The family did not ask questions. Instead, they waited for Dewi to elaborate. In the corner of the room, while making my notes, I also wondered what the psychologist was implying: not being cured but being “healthy”? And why did she say this when she knew the patient's condition was declining? Is there any potential healthiness when death is imminent?

After the pause, Dewi elaborated by saying that cancer is already in the body, and palliative care aims to treat the symptoms rather than cure the disease. She also stated:

“We do our best so that patients feel comfortable and not in pain. If we think about a cure, we will feel… [did not complete the sentence]. So, now we focus on what we can do…”

As we see from the excerpts above, palliative care professionals carefully choose their language to avoid using terms such as death and dying, instead shifting their narrative to focus on care. When moving the narrative away from curative treatment to non‐curative care, Dr Stefani navigates the tension by building on a palliative‐care rhetoric that focuses on comfort and quality of life and preparing patients for care post‐discharge. However, these are not terms that are necessarily understood by families, as the jargon of “comfort” (*nyaman*) and “quality of life” (*kualitas hidup*) are not often used in Indonesia. This is what led Dewi to try to clarify by making a seemingly paradoxical statement that the family should not focus on a cure (*sembuh*), but on health.

In everyday life, the term *sembuh* is often used to wish someone well when the person is ill—“*semoga lekas sembuh*” means literally “get cured soon.” However, as *s* *embuh* or cured means a state of being free from disease or illness, it can, perhaps surprisingly, also imply that the patient has died. When someone passes away, people often say: “The deceased is now *sembuh*” as the individual is now free from illness.

Unlike *sembuh* (cured), *sehat* (healthy) does not merely denote the absence of disease. Slightly different from the conceptualization of *sehat* (healthy) from the World Health Organization's 1946 constitution, which defines health as a state of complete physical, mental, and social well‐being, in one of my interviews with Dewi, she explained that in Indonesia, there are two types of health that are intertwined: *sehat raga* (a healthy body), which is related to the physical and material wellbeing, and *sehat jiwa* (a healthy soul), which is closely related to not only psychological and emotional but also spiritual wellbeing (the latter of which is not included in the WHO definition of health). By invoking the concept of *sehat* in this context, Dewi reminded the family of the patient's spirituality—particularly the importance of having peace of mind and a sense of meaning in life (see also Choong, 2015). In this way, letting go of curative treatment and focusing on being healthy implies that the family should focus on supporting the patient's spiritual connection with God during illness and adjusting their expectation with regard to a cure, which is what they were aiming for when they came to the hospital. Emphasizing the aim of being healthy, despite not being cured, therefore shows sensitivity in subtly discussing end‐of‐life care with the family.

More practically, to help family members navigate care at home, the palliative care unit also provides a hotline service. Patients and family members are allowed to ask questions if there is confusion about care at home, including what to do when the patient's condition is deteriorating. Palliative care nurses usually close the family meeting with a reassuring narrative, exemplified by what Nina, the nurse, told Yusuf and his children at the end of the meeting about Emi:

“You do not have to worry. We will not leave you alone. After the patient is discharged from the hospital, you can reach us through the palliative care hotline. If you are confused at home after hospital discharge, please let us know through our WhatsApp number. You do not have to bring the patient immediately to the emergency department.”

While offering an extension of care through the hotline, Nina's advice to not take the patient back to the hospital also implies that she will no longer receive curative treatment. In this way, the palliative care team also tries to prevent patients from dying in the hospital (or on the way to it). Palliative care professionals told me they were driven by the idea of the patient dying at home and being close to the family to create a ‘good death’ for the patient. Crucially, the palliative care professionals felt they were not doing a good job if the patient and the family, having been referred to the palliative care unit for a hospital discharge meeting, returns to the emergency department for treatment. The death of such patients in the hospital is considered a failure.

Despite this, family members often panic post‐discharge, especially when patients are in pain or their condition deteriorates. In Indonesia, there is hardly any public institutional support which focuses on palliative care or pain management at home, and there is a lack of access to pain medication (see also Putranto et al., 2017). Thus, family members still often take patients to the hospital's emergency department, even though they have been told not to do so.

At the end of the meeting, Yusuf said that he appreciated the care, attention, and support of the palliative care team. Thanking them, he said:

“We accept the situation. It is *taqdir* (predestination). But it is all in God's hands, right?” (He smiled)

The meeting was wrapped up by everyone shaking hands. Saying that everything is in God's hands is a polite and normative way to end this sort of meeting. In this particular context, however, it also emphasized the open‐endedness of the future, with Emi's husband both confirming that he had understood the message that the care team had just delivered regarding the seriousness of Emi's condition, and yet also concurring that a future cure was not completely impossible, because ultimately it was in God's hands. Hearing this acknowledgement that Yusuf had accepted Emi's condition gave the palliative care team a sense that the meeting had gone well and that they had achieved their goal. There was no conflict, the family was informed about the patient's condition, and the tensions had been resolved.

## CONCLUSION

Palliative care professionals who are mandated to assist with the discharge process for patients who can no longer receive curative treatment carefully navigate the tensions and confusion of this process by employing implicit, ambiguous, and culturally sensitive communication to discuss end‐of‐life care with the patient's relatives. As I have shown in this paper, palliative care professionals in the tertiary hospital in Indonesia “care‐fully” disclose information about prognosis and end‐of‐life care in a respectful way by using terms such as *belum* (not yet) to keep the possibility of treatment open, and *sembuh* (cured) and *sehat* (healthy) to shift the narrative from cure to care. Similar to Black's (2015) findings in South Africa, in this Indonesian palliative care setting, the use of implicit communication strategies can be a means of navigating a “morally conflicting situation.” Such strategies help palliative care professionals to maintain the balance between disclosing prognostic information, managing patients’ and families’ hopes for a cure, and adhering to institutional mandates to assist the discharge process. Based on this, I suggest that these communicative practices enable a more nuanced, respectful, and ethically sensitive approach to hospital discharge and end‐of‐life care, particularly in a context where direct discussion of end of life is considered potentially harmful.

The use of terms such as *belum* (not yet) to keep the possibility of treatment open, and *sembuh* (cured) and *sehat* (healthy) to shift the narrative from cure to care, illustrates how palliative care professionals carefully attend to both the referential and the performative dimensions of the language they use. This resonates with Kimberley Arkin's (2020) findings in southern France, where, in their treatment of a Muslim patient, healthcare professionals chose specific language and carefully avoided the word prognosis, as it could be a potentially dangerous performative act. Slightly different from the physicians in Arkin's study, however, for Dr Stefani and her colleagues, using the word *belum* is not only a performative act but also refers to a shared cultural/religious concept of not ruling out possibilities because biomedicine is seen as not ultimately able to predict the time of death. In this setting, healthcare professionals do not just choose their words performatively, considerate of patients’ cultural and religious contexts, as many healthcare professionals themselves share similar values and beliefs. In terms of death and dying, there is often a deep sense of “existential ambivalence” as to whether a patient is dying and how long they will live (see Flaherty, 2018). For some of the physicians reluctant to discuss prognosis, such as Dr Ani, there is a truth in the use of *belum* from a religious viewpoint. From a religious perspective, there could always be a miraculous healing intervention. Even if healthcare workers do not expect such a miracle to take place, they may maintain this ambiguity by using *belum* out of sensitivity to the faith of the patient and the patient's family.

In the context of the rapidly growing diversity of palliative care initiatives around the globe and the rising medicalization of death, the professional practices that I observed show that palliative care professionals are creatively developing and defining palliative care in a particular cultural and institutional context (see also Samuels & Lemos Dekker, 2023; Zaman et al., 2017). Palliative care practices are shaped to fit the social and cultural setting as well as healthcare structure, incorporating Islamic and culturally specific religious values—elements that have yet to be fully integrated into the global palliative care models (see also Al‐Wamer & Downar, 2014). These practices include pluralistic approaches to discussing end‐of‐life care. Attuned listening to the implicit and embodied communication in diverse palliative care contexts, particularly in the Global South, offers nuanced insight into the plurality of culturally sensitive approaches to “care‐full” conversations in the last phase of life.

## ENDNOTES

1 All individuals referred to in this paper have been given pseudonyms.

^2^
Throughout this article, I use end‐of‐life as an adjective when referring to care (“end‐of‐life care”) and otherwise, without hyphens, as a noun (“end of life”).

3 According to the World Population Review (2025), Indonesia's Muslim population is approximately 242 million.

4 This is most common among those patients reliant on public healthcare or public insurance but may not be the case if someone can pay for private healthcare because they may be able to stay for as long as they wish (see e.g., Banerjee, 2022).

5 This observation should not be taken as a universal representation of all Muslim patients, as individual experiences vary according to school of thought and degree of religious observance, as well as individual circumstances.

6 Ethical approval was granted by the university's ethics committee, the local ethics committee at the partnering university in Indonesia, and the ethics committee of the hospital where the study was conducted.
